# Dysfunctional Glucose Metabolism in Alzheimer’s Disease Onset and Potential Pharmacological Interventions

**DOI:** 10.3390/ijms23179540

**Published:** 2022-08-23

**Authors:** Vijay Kumar, So-Hyeon Kim, Kausik Bishayee

**Affiliations:** 1 Department of Biochemistry, Institute of Cell Differentiation and Aging, College of Medicine, Hallym University, Chuncheon 24252, Korea; 2 Biomedical Science Core-Facility, Soonchunhyang Institute of Medi-Bio Science, Soonchunhyang University, Cheonan 31151, Korea

**Keywords:** Alzheimer’s disease, diabetes, glycolysis, ROS, genetic mutation, therapy

## Abstract

Alzheimer’s disease (AD) is the most common age-related dementia. The alteration in metabolic characteristics determines the prognosis. Patients at risk show reduced glucose uptake in the brain. Additionally, type 2 diabetes mellitus increases the risk of AD with increasing age. Therefore, changes in glucose uptake in the cerebral cortex may predict the histopathological diagnosis of AD. The shifts in glucose uptake and metabolism, insulin resistance, oxidative stress, and abnormal autophagy advance the pathogenesis of AD syndrome. Here, we summarize the role of altered glucose metabolism in type 2 diabetes for AD prognosis. Additionally, we discuss diagnosis and potential pharmacological interventions for glucose metabolism defects in AD to encourage the development of novel therapeutic methods.

## 1. Introduction

Alzheimer’s disease (AD), the most generic form of dementia, is an irreversible, progressive brain disorder that destroys neuronal cells. AD is the fifth leading cause of death for people aged sixty-five and older [1]. Scientists do not yet fully understand the cause of this disease, which is likely to involve several factors and can affect each person differently. Health care providers often fail to diagnose AD at an early stage; thus, researchers are currently working on a diagnostic framework in which AD onset can be detected based on biological changes in the brain and body even before any symptoms appear [2]. Early AD identification remains challenging as the conventional biomarkers for AD can overlap with the classical aging process. Scientists have grouped the AD biomarkers into the ATN framework, where A is amyloid, T is phosphorylated tau, and N is neurodegeneration [3]. Markers such as amyloid-β (Aβ) plaques and tau tangles are well-known indicators of AD; however, these proteins are often present at a higher physiological level in older adults [4,5,6].

Recent studies suggest that dysfunctional glucose metabolism is often found in AD brains. An aged-matched comparison between regular and AD brains showed reduced glucose utilization, evidenced in APP (AD model) mice [7]. Thus, glucose utilization could be an early important imaging marker for AD detection. Under normal physiological conditions, brain cells use a relatively higher percentage of glucose for their function and energy source [8]. Alteration in cerebral glucose metabolic rate and glucose consumption are reflected in the synaptic excitability and neuronal activity [9]. In the AD brain, a lesser extent of glucose utilization was detected by positron emission tomography (PET) using 18F-fluorodeoxyglucose (FDG) as a tracer [10]. Specifically, a reduction in glucose consumption at the hippocampal and posterior cingulate was observed in the early AD stages [11,12,13]. Later, in the advanced stages, glucose consumption reduces at the temporal–parietal cortex and the frontal and occipital cortices [12]. The decline in glucose metabolism is, moreover, correlative with synaptic density and function [14,15], suggesting that cognitive impairment has a connection to glucose consumption in the brain. The reduction in glucose consumption is directly linked with type 2 diabetes mellitus (T2DM), characterized by insulin resistance and chronic systemic inflammation. Now scientists are concentrating on the relationship between T2DM and the development of AD syndrome in the later stages of life. This review describes the pathogenesis due to glucose restraint and the development of cognitive dysfunctions in the brain during AD onset.

## 2. Cerebral Glucose Metabolism

**Figure 1:**
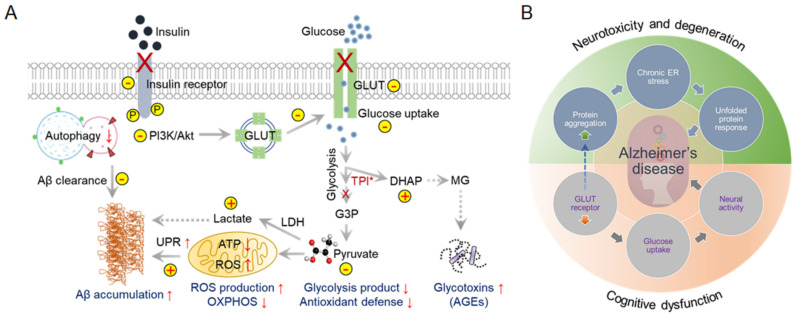
Dysfunctional cerebral glucose metabolism is a crucial factor in AD development. Glucose catabolism in AD is directly connected to further negative consequences in AD syndrome development. (**A**) Schematic representation of defective glucose metabolism in AD and various outcomes. ↑ arrows indicate upregulation and ↓ arrows indicate downregulation. (**B**) Glucose metabolism forms the bridge between neurotoxicity and cognitive dysfunction in AD. This interconnected Venn diagram describes the scenarios in which AD-related toxicity and cognitive dysfunction appear during AD onset and progression.

AD patients show reduced glucose metabolism in the brain. Studies have shown that improved glucose uptake and consumption have neuroprotective effects, including increased proteostasis [16]. Dysregulated glycolytic enzymes reduce glucose utilization and, alternatively, by increasing the protein glycosylation process, induce age-related neurodegeneration [17,18]. Moreover, cerebral glucose uptake in the aged brain reduces due to the reduction in the expression of the important glucose transporters, GLUTs. For example, neuronal GLUT3 and GLUT4 expression is significantly reduced due to the lower insulin responsiveness in the T2DM brain. In addition, the glycolytic enzyme triose-phosphate isomerase (TPI) plays a crucial role in neurological dysfunction due to mutations [19]. Dysfunctional TPI is responsible for reducing the NADH content. Insufficient NADH generation reflects mitochondrial hypometabolism in TgAD mice [20]. Alternatively, inactivated TPI is also responsible for the accumulation of dihydroxyacetone phosphate (DHAP), which decomposes to methylglyoxal (MG) and is responsible for the accumulation of advanced glycation end-products (AGEs) in AD patients’ brains (Figure 1A) [21].

In addition to the TPI abnormalities, the lactate dehydrogenase (LDH) activity level increases in aged brains. LDH reduces pyruvate concentration by catalyzing pyruvate to lactate in neurons [22,23,24]. A reduction in pyruvate levels alters the antioxidant defense mechanism and oxidative phosphorylation (OXPHOS) in mitochondria [25,26], triggering oxidative stress and a misfolding protein response. Abnormal glycolytic pathways in the brain could therefore be an early hallmark of AD (Figure 1B). Impairment of the default glucose catabolic process can lead to Aβ deposition or Tau protein phosphorylation [27]. Thus, targeting metabolic reprogramming in neurons is a novel approach for AD intervention and treatment.

## 3. Monitoring Glucose Uptake and Metabolism in AD

The human brain uses about 20% to 25% of the total glucose utilization in the body to perform synaptic activity. Principally, the alterations in glucose utilization practically hinder natural cellular functions, including synaptic functions in the brain. Typically, insulin receptors in brain cells control the process of glucose consumption and metabolism. Thus, the development of insulin resistance significantly raises the risk of AD [28,29]; additionally, T2DM increases the risk of AD by 50% [30,31].

**Figure 2:**
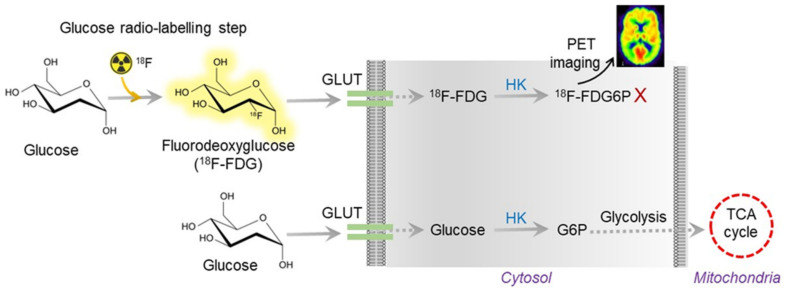
Overview of 18F-FDG PET working mechanism. The radioactive FDG is converted to FDG6P, which can be measured and quantified by PET imaging. In AD, the glucose uptake is vastly reduced due to insulin resistance. Thus, the radioactive tracer does not show up in AD brain imaging.

In the AD brain, a lesser extent of glucose utilization was detected by positron emission tomography (PET) using 18F-fluorodeoxyglucose (FDG) as a tracer, which is a radiolabeled sugar (glucose) molecule (Figure 2). Imaging with 18F-FDG PET was used to determine the sites of abnormal glucose metabolism [32]. This detection method is now extensively used to locate tumors and sites of recurrent disease in cancer patients (ClinicalTrials.gov Identifier: NCT00207298). The use of 18F-FDG PET imaging is growing because a decrease in glucose utilization correlates with the appearance of cognitive symptoms in AD [33]. Thus, 18F-FDG PET is now considered a practical and independent biomarker for examining the metabolism changes in the pre-symptomatic phase of AD [34]. However, the use of 18F-FDG PET is still limited due to its high cost and limited availability in general clinics around the globe. Moreover, the radioactive material is unsuitable for repeated measurements in patients, even though it has high specificity and sensitivity.

As an alternative, magnetic resonance imaging (MRI) is receiving attention for monitoring sugars in tumor samples [35]. MRI scans can identify brain malformations connected with mild cognitive impairment (MCI) and predict which patients with MCI may eventually develop AD. Nontoxic glucose can be sensed and imaged by dynamic glucose-enhanced (DGE) MRI. DEG-MRI can identify the brain’s glucose distribution, transport, uptake, and metabolic rate [36]. Recent animal studies have demonstrated that d-glucose is a biodegradable MRI contrast agent for imaging glucose uptake in tumors [35,37]. Additionally, d-glucose can potentially cross the blood–brain barrier (BBB), making DGE-MRI feasible for studying glucose uptake and metabolism in the human brain [38]. Experiments involving WT mice and age-matched APP/PS1 transgenic AD mice demonstrated a notable reduction in signal intensity for d-glucose uptake in the brain parenchyma and CSF. Reduced glucose signals could result from impaired glucose uptake in the AD brain due to the reduction in GLUT1 and GLUT3 expression at BBB [7]. Recently, Huang et al. modified the DGE-MRI to detect D-glucose delivery in the brain [7] and identified altered glucose uptake and clearance in young AD mice before the emergence of amyloid plaques. Another widely explored imaging technique in cancer metabolism is chemical exchange saturation transfer (CEST) MRI [39,40]. Scientists used CEST-MRI to detect 2-deoxy-D-glucose (2DG) uptake in AD mouse (APP23 model) brain in different regions of the brain [41]. CEST technique can distinguish the decline in 2DG brain uptake in 20-month-old APP23 mice compared to WT mice. The brain glucose metabolic activity differences can be detected within a few minutes of injecting 2DG into the cortex area [42]. It is necessary to employ a 3T clinical field strength and use nontoxic sugars to expedite the conversion of DEG-MRI or CEST-MRI to clinical AD diagnosis.

## 4. AD Onset and Pathogenesis

**Figure 3:**
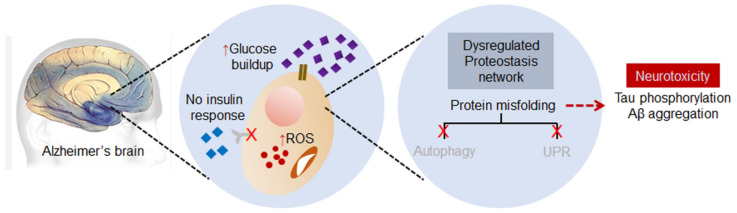
Dysregulated proteostasis network leads to neurotoxicity in AD brains. The UPR system usually ceases, and the buildup of aggregated Aβ plaques and additional misfolded proteins often induces cell death signals in the neurons. ↑ arrows indicate upregulation.

The understanding of AD onset is an essential factor in designing an efficient therapeutic method. AD is a highly progressive and complex neurodegenerative disorder and a major cause of dementia in old age. Although AD onset at a younger age is uncommon, there is growing evidence that AD onset can be observed at a younger age [43,44]. The primary cause of AD remains the accumulation of aggregated Aβ plaques and neurofibrillary tangles (NFTs). The development of these aggregates initially disperses throughout the cortex region (basal, temporal, and orbitofrontal). However, at the later or aggressive stages these tangles can be detected in several other parts of the brain, such as the cortex, hippocampus, amygdala, diencephalon, and basal ganglia regions [6,45]. Scientists previously believed that the deposition of NFTs in the brain alters the neuroinflammatory markers. However, factors such as type 2 diabetes mellitus (T2DM) linked with higher glucose levels have been considered in the induction and amplification of neuroinflammation in the AD brain [46,47,48]. The association between low glucose utilization in the brain and dementia could imply several aspects, such as insulin resistance, protein glycosylation and misfolding, and the accumulation of oxidative stress products (Figure 3).

On the other hand, oxidative stress-mediated toxicity is an undisputed cause of and link between AD and T2DM. The oxidized proteins and lipid products can be used as potential disease progression markers [49]. A decrease in glucose uptake in AD and T2DM slows down the glucose catabolic process and reduces antioxidant pyruvate levels [25,26]. Moreover, the accumulation of Aβ plaques was reported as an inducer of oxidative stress and protein misfolding-related stress (ER stress) via the impairing of mitochondrial redox potential [50]. Alternatively, ROS accumulation increases abnormal tau phosphorylation via glycogen synthase kinase 3 (GSK3) [51] and increases the apoptosis signal-regulating kinase 1 (ASK1)–p38 MAPK axis in AD brain aging [52,53]. Unutilized glucose accumulation due to the unresponsive insulin receptor (in T2DM) contributes to hyperglycemia and develops various cytotoxic complications. Hyperglycemic protein misfolding is a common issue with T2DM [54], and misfolded protein deposits composed of Aβ and tau trigger dysfunctional proteostasis in AD [55]. Unfolded/misfolded protein response (UPR) activation was observed in postmortem brains of AD [56]. Moreover, the suppression of the ER stress-related proteins PERK and eIF2a can reverse the de novo protein synthesis deficits and memory loss in AD transgenic mice [57].

**Figure 4:**
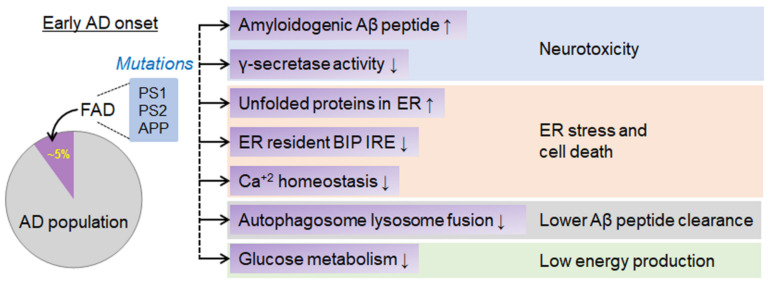
The early onset of AD at a younger age is due to the mutations in lysosomal membrane proteins. In these conditions, standard autophagy clearance is hampered. Primary research identified various other signaling mechanisms in FAD patients. ↑ arrows indicate upregulation and ↓ arrows indicate downregulation process.

About 5% of AD cases are due to genetic mutations transmitted through families, otherwise known as familial Alzheimer’s disease (FAD) [58]. In these cases, the affected persons usually develop symptoms at an earlier age, well before age 65, and symptoms occasionally start as early as in their 30 s or 40 s [58]. This condition arises due to the mutations in the APP, presenilin-1 (PS1), and presenilin-2 (PS2) [58,59]. Mutations in these genes alter the proteolytic processing of APP, such as an increase in the production of the highly amyloidogenic Aβ peptide. Both PS1 and PS2 are crucial in the γ-secretase-mediated cleavage process in generating Aβ [60]. Lysosomal membrane protein (PS1/PS2) double knockout mice lack γ-secretase activity apart from membrane trafficking [61,62]. Mutations in PS1 change the UPR, which increases the number of unfolded proteins in ER and causes ER stress [63]. PS1 mutations also decrease chaperone BIP and ER-resident transmembrane kinase and IRE1 levels, responsible for protein folding and sensing misfolding [64]. Data suggest that reduced BIP and IRE1 levels were observed in AD brains. Additionally, in PS1/PS2 null neurons, autophagy cannot clear amyloidogenic Aβ peptides. Autophagosome and lysosome fusion is inhibited in PS1/PS2 null cells [65]. Moreover, presenilin (PS) maintains Ca^2+^ homeostasis in the cytosol, mitochondria, and endoplasmic reticulum [66]. Aberrant changes in calcium signaling diminish the capacity to manage oxidative stress, induce ER stress, promote the accumulation of Aβ plaques, reduce synaptic transmission, and reduce neuronal viability in AD [67,68,69,70,71] (Figure 4).

The PS1 mutation possesses glucose metabolism defects. In PS1 His163Tyr mutation carriers, statistical parametric mapping (SPM) showed lower thalamic cerebral glucose metabolism at younger ages [72]. In another instance, the PS1 Met146Val mutation showed a global cortical glucose hypometabolism [73]. Moreover, PS2 mutant overexpression reduced glucose usage and enhanced Aβ-42 peptides in mice [74]. The presenilin/Notch1 axis can also trigger several microRNAs, including miR-375, miR-30a, and miR-34a, at the downstream signaling process [75]. miR375 is a known inducer of cell death in neuronal cells [76]. Therefore, presenilin is a connecting link between several essential signaling pathways in AD, including glucose hypometabolism, Ca^2+^ signaling, and the induction of ER-related stresses.

## 5. Type 2 Diabetes and AD Link

Epidemiological studies have entrenched the initial connection between the pathogenesis of T2DM and AD. Repeated evidence has shown that T2DM patients have a higher risk of AD, mainly in the aged population [77,78]. Several studies have suggested that diabetes can increase the risk of AD by approximately 50% in the above 60 years age group [30,79,80]. However, recent studies established several shared links between T2DM and AD, including insulin resistance, oxidative stress, and genetic factors [81]. This section briefly summarizes the shared links frequently associated with T2DM and AD.

### 5.1. Insulin Resistance

**Figure 5:**
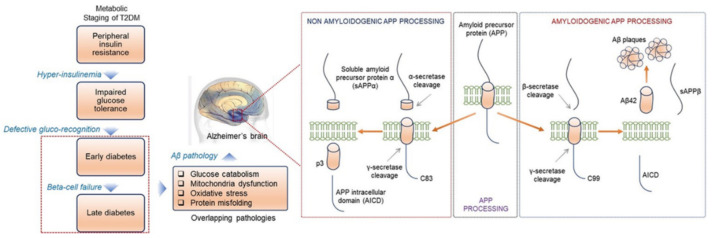
Proteolytic cleavage of APP. In the non-amyloidogenic pathway, the extracellular domain cleavages by α-secretase and releases the soluble peptide sAPPα. In contrast, the amyloidogenic pathway releases Aβ42, a significant component of Aβ plaques.

Insulin is a pancreatic peptide hormone regulating cells’ overall glucose movement or absorption. Additionally, insulin acts as a signal and activates the signaling by membrane-bound insulin receptors (IR), which play an essential role in neuronal growth and differentiation and the release of neurotransmitters [82]. In T2DM individuals, cells have reduced insulin sensitivity, frequently termed insulin resistance. Particularly in the brain, this phenomenon is known as diabetes type 3 (brain diabetes) [83] and is an emerging cause of AD pathogenesis [28,77,78,82,84]. At the cellular level, decreased insulin action may result from impaired and deficient insulin receptors (IR) or impaired intracellular signaling [85]. The important AD-causing factors include the accumulation of amyloid-β (Aβ) plaques, Tau protein hyperphosphorylation, and inflammation linked to insulin resistance. Amyloid beta (Aβ) is the product of proteolytic cleavage of the β-amyloid precursor protein (APP), which is essential for synaptic plasticity, neuronal activity, and memory. APP is a single domain membrane integral protein. It has a long glycosylated extracellular N-terminal and short cytoplasmic C-terminal domain (Figure 5). APP is also involved in synaptic formation, anterograde neuronal transport, and iron export [86].

The proteolytic cleavage of APP occurred in two different pathways—first, the non-amyloidogenic pathway and, second, the known amyloidogenic pathway (Figure 5). Insulin promotes the non-amyloidogenic route of APP processing; therefore, deficiency/impairment of insulin signaling triggers alternate (amyloidogenic) processing and accretion of Aβ toxicity [82,87]. Insulin and Aβ are the substrates of an insulin-degrading enzyme (IDE), and insulin resistance reduces the expression of IDE and Aβ degradation, leading to AD pathology [88]. The tau proteins are considered another vital factor for AD pathogenesis. Tau proteins are the six (soluble) splicing variants of the microtubule-associated protein tau (*MAPT*) gene, highly expressed in neurons of the central nervous system (CNS) [89]. In a healthy neural cell, tau proteins bind to microtubules (MT) and modulate the stability (Figure 5). However, tau is also reported to recruit the signaling molecules and MT-mediated axonal transport [90]. Tau itself contains 85 putative phosphorylable residues (serine, threonine, and tyrosine) [91] that are tightly regulated by a balanced action of several kinases and phosphatases [92]. Hyperphosphorylated tau protein forms the neurofibrillary tangles (NFT), which is an aggregated form of tau proteins (Figure 5) [78]. More than 30 sites are known to be a substrate of GSK-3β, which suggests that the insulin or insulin-like growth factor 1 (IGF-1) may contribute to AD pathogenesis and NFT toxicity [85,93]. In insulin-deficient mice, changes in GSK-3β activity by streptozotocin (STZ) can alter tau phosphorylation. The hyperphosphorylation status of tau protein was moderate within 30 days of treatment [92,94] and was highest after 40 days [94]. Moreover, tau phosphorylation status correlates with GSK-3β activity in neuroblastoma cells [95]. Alternatively, tau phosphatase (protein phosphatase 2A), an enzyme that inhibits the phosphorylation of tau, was down-regulated in STZ-treated mice [96]. Furthermore, a recent study reported the overexpression of a cancerous inhibitor of PP2A (CIP2A; an endogenous inhibitor of PP2A) in the AD brain that allows tau phosphorylation and drives the AD pathogenesis [97]. The evidence suggests that insulin resistance/deficiency might directly affect AD; however, the detailed molecular mechanism requires future investigation.

### 5.2. Oxidative Stress

Normal cells maintain cellular homeostasis by balancing reactive oxygen species (ROS), reactive nitrogen species (RNS), and antioxidants. Impaired balance causes oxidative stress, leading to protein/DNA damage, cellular injury, and compromised cell integrity that causes cell death. Approximately 5% of the total produced oxygen generates ROS, which play an essential role in normal physiological processes, including signaling, cell proliferation, and survival. At the cellular level, the accumulation of oxidative stress was reported in both diabetes and AD patients [98]. In diabetic patients, hyperglycemia triggers mitochondrial dysfunction by reducing the functional activity of glyceraldehyde 3-phosphate dehydrogenase (GAPDH; a critical glycolytic enzyme) [30,31,99]. Simultaneously, the impaired metabolism of the neural mitochondria slows down adenosine triphosphate (ATP) production, inducing ROS generation, the deposition of extracellular Aβ, and tau proteins [100]. However, the exact molecular mechanism underlying oxidative stress or the dysfunction of mitochondria, and whether it plays a direct role in AD initiation, has not been fully elucidated. Here, we review some key evidence suggesting that T2DM and AD are integrated events in an imbalanced redox state. It has been reported that oxidative stress in mitochondria damages the mitochondrial encoding gene (subunit of electron transfer chain), leading to less ATP production and compromising calcium homeostasis [101,102]. Simultaneously, higher activity of JNK/P38 MAPK, elevated ROS, aggregation of Aβ, and phosphorylation of tau protein have been reported in human and other vertebrate models [100,103,104]. Similarly, other reports demonstrate that superoxide and thiobarbituric acid reactive species (TBARS) accumulate in the prefrontal cortex and hippocampus regions of diabetic rats [105], which may elevate AD pathogenesis.

Moreover, the activity of antioxidant enzymes, such as superoxide dismutase (SOD) and catalase (CAT), reduces in the amygdala and prefrontal cortex of diabetic rats [106]. A reduction in ROS-scavenging enzymes, namely SOD, CAT, and glutathione peroxidase (GPx), may elevate the oxidative stress in the brain, which may induce AD progression [107]. Additionally, SOD1 knockout induces oxidative damage followed by Aβ oligomerization and Tau phosphorylation [108,109]. Similar evidence was reported in AD patients; the oxidized protein and lipid levels significantly increased [108]. The endoplasmic reticulum (ER) is an important site for generating the amyloidogenic species of Aβ [110]. Accumulating Aβ in ER hampered several cellular processes, including calcium homeostasis, which further promote AD progression or cell death [100]. In antioxidant defense systems, erythroid 2-related factor (Nrf2) is an essential nuclear factor that regulates several oxidative stress-related genes [111]. Previously, it has been reported that inactivation of Nrf2 significantly elevates the oxidative stress consequence of aging; however, Nrf2 gain-of-function restored cellular homeostasis and viability [100,111,112]. Altogether, this evidence suggests that dysfunctional mitochondria, impaired cellular homeostasis, and oxidative stress are common characteristics that may induce diabetes and AD progression, or vice versa.

### 5.3. Genetic Factors

Extensive studies reported that T2DM is a risk factor for AD pathology; however, the molecular basis underlying the common genetic association between T2DM and AD remains elusive. Hokama et al. analyzed the dysregulated gene expression in brain tissues of AD patients and reported that diabetes-related genes were significantly altered in the hippocampus [113]. A comparative RNA sequencing of AD and diabetes patients showed similar molecular signature networks, which included phosphatidylinositol 3-kinase (PI3K) and protein kinase B/Akt (PKB/AKT) pathways involving insulin signaling [114]. Simultaneously, transcriptomic meta-analysis data identified the dysregulation in the forkhead box O3 (FOXO3) transcription factor [114]. Further, Lee et al. identified five transcription factor genes, *COPS4*, *PSMA6*, *GTF2B*, *GTF2F2*, and *SSB*, which are common in both T2DM and AD [115]. In the shared genetic network in T2DM and AD, Hu et al. developed novel causal inference methods [116]. Based on 448 individuals (ROSMAP project), the pipeline identified 13 genes and 16 pathways common between T2DM and AD [116]. Similarly, Chung et al. used the non-negative matrix factorization (NMF) method of meta-analysis and identified 241 genes that were common between T2DM and AD [117]. Several single nucleotide polymorphisms (SNP) genes were also identified as risk factors for T2DM and AD pathology [96]. This evidence indicates that T2DM and AD may share genetic factors, although future investigation and biochemical evidence will provide a clearer picture.

## 6. Epidemiology and Treatment of AD and Type 2 Diabetics

Over 55 million people have dementia worldwide, with AD accounting for approximately 70% of these cases. The number of affected people is estimated to rise by 139 million by 2050 [118,119], indicating the urgent requirement for effective treatment. Similarly, there are 536.6 million diabetic people worldwide in 2021, estimated to increase to 642.7 million by 2030 [120]. Scientific achievements have prolonged the lifespan of diabetes patients with market-accessible medicine or hospital care [121,122,123,124]. In this section, we briefly review the progress made in AD treatment. Unfortunately, there is no treatment for AD; more attention must be paid to the associated metabolic diseases, including diabetes. Therefore, discovering new associated factors is a reasonable assurance for AD prevention. New pharmacological targets, as well as antidiabetic medications, are being investigated for AD treatment. Recently, metformin has been under clinical trials (Phase III/IV) for AD patients with mild cognitive dysfunction (Table 1).

**Figure 6:**
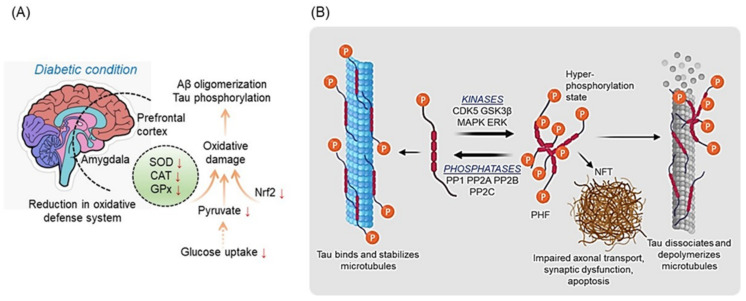
(**A**) The inhibition in the oxidative defense system potentiates Aβ oligomerization and Tau phosphorylation in the diabetic brain. ↓ arrows indicate downregulation. (**B**) Tau phosphorylation and NFT formation. In a healthy cell, phosphorylated tau protein binds with microtubules (MT) and maintains stability. However, hyperphosphorylation tau aggregates into the NFTs which leads to MT instability. This figure was drawn using BioRender application (https://biorender.com (accessed on 21 June 2022)).

Histopathological studies revealed the accumulation of NFTs of tau protein and Aβ plaques in the AD brain, which is considered the prime target for drug development. Currently, acetylcholinesterase inhibitors (donepezil, rivastigmine, and galantamine) are used as drugs for improving cognitive impairment (Table 2). It can inhibit the hydrolysis of acetylcholine in the synaptic cleft [125,126]. To treat moderate to severe AD, clinicians also use the NMDA receptor antagonist memantine (Table 2). Memantine activates the PP2A, reduces tau hyperphosphorylation, and regulates Ca^2+^ influx that neutralizes glutamate-induced excitotoxicity [125,126,127]. This compound is not a proper cure for AD, although it improves the patient’s cognitive functions and performance. Several natural or synthetic small compounds and peptides (or peptidomimetics) are being studied as a direct or indirect target against Aβ aggregation. Targeting the APP processing enzymes, including β- and γ-secretase (Figure 6), might prevent de novo Aβ deposition, which could improve the brain’s health. The small brain-permeable molecules that inhibit β-secretase (or β-site APP cleaving enzyme1 (BACE1)) include verubecestat, lanabecestat, and LY3202626, and have been shown to cause a significant reduction in de novo Aβ formation during initial trial phases. However, they failed in clinical trials and the FDA approval process due to their adverse side effects, psychiatric issues, and futility [126].

On the other hand, several types of γ-secretase inhibitors, for example semagacestat and avagacestat, showed promising results and decreased the production of Aβ in AD patients [125,126,128,129,130]. Jeffrey et al. found that a total of 143 different drugs are currently under clinical trials for AD treatment (based on January 2022 U.S government (clinicaltrials.gov (accessed on 23 June 2022)) data) (see the cited article for details [131]).

Recently, aducanumab became the first approved anti-amyloid monoclonal antibody for AD treatment. Donanemab and lecanemab are other monoclonal antibodies approved by the FDA (Table 2) [132,133]. These modern monoclonal antibody-based therapies may provide relief in delaying or preventing AD. Similar monoclonal antibody-based therapies are now in the late stages of clinical trials (Table 1). With the current progress made in drug development by targeting Aβ, tau, and immune system activation, we refer the reader to the latest cited articles [125,126,127,131,132,134,135].

## 7. Future Directions

Several previous clinical trials focusing on the Aβ cascade failed to pass the clinical approval stages, with a failure rate of more than 99.6% [136]. The current strategies targeting Aβ and tau are unlikely to form the future cure for AD. Therefore, more effective and accessible therapies are urgently needed. Scientists need to reconsider several crucial factors for developing therapeutic strategies. Nonetheless, while considering metabolic analytical data, we find that glucose metabolic alterations could be a prolific target for developing next-generation drug candidates for AD therapy. In this review, we highlighted how altered glucose uptake induces diabetic-type conditions, and several lethal downstream pathways trigger neuronal cell death, thus inducing cognitive impairments. Niccoli et al. showed that overexpression of the Glut1 receptor rescued Drosophila in the Aβ model; moreover, they showed that metformin treatment helps flies to recover from Aβ toxicity by elevating UPR responses [137]. T2DM involving insulin resistance is considered a risk factor in developing AD. The “homeostatic model of peripheral insulin resistance” (HOMA-IR) has shown worse performance in memory factor scores [138]. In addition, reduced GLUT1 expression at the blood–brain barrier worsens AD-related neurodegeneration and cognitive function [139]. Therefore, we consider a reduction in cerebral glucose uptake to be an early and accurate biomarker of dysfunction in AD. Though plenty of research is going on in the field of AD and T2DM, clinicians are still not sure of the exact reason behind AD’s more common occurrence in T2DM patients. People with diabetes may have a slow or blocked blood flow in the brain due to injured blood vessels, which also can lead to vascular dementia. One way to slow down brain decline is to keep diabetes under control with medicine, exercise, and a healthy diet. The high heterogenicity between different cases of AD and T2DM with aging makes it more challenging to dissect the specific mechanisms responsible for the general association of AD and T2DM observed in the population. Therefore, we must be more cautious while proposing specific mechanisms or treatments.

## Figures and Tables

**Table 1:** Late-stage clinical drug development (phase II/III, III, and IV clinical).

Class	Drug Name	Molecular Target	Recruited Population	Project Number/Reference
Amyloid targeted	Crenezumab	Anti-amyloid β antibody	Genetic mutation carriers	5R01AG055444-04
	BAN2401	Anti-amyloid β antibody	Intermediate amyloid level (by screening PET)	5R01AG054029-03
	Solanezumab	Anti-amyloid β antibody	Amyloid positive (by brain imaging)	5R01AG063689-03
	BAN2401	Anti-amyloid β antibody	Elevated amyloid levels (by screening PET)	5R01AG061848-02
	ALZ-801	Tramiprosate pro-drug	Individuals with APOE4/4 and early AD diagnosis	5R01AG065253-02
Metabolism	Metformin	amyloid β	Mild cognitive impairment	5R01AG062624-03
Synaptic Plasticity	AGB101 (levetiracetam)	Synaptic vesicle protein (SV2A) modulator	Amnestic mild cognitive impairment	5R01AG061091-04

**Table 2:** The FDA-approved drugs for AD treatment (ARIA; amyloid-related imaging abnormalities).

Drug Name	Target	Common Side Effects
Aducanumab	Monoclonal antibody for aggregated Aβ	Headache, dizziness, falls, ARIA, confusion
Donanemab	Monoclonal antibody for aggregated Aβ	Chills, dizziness, flushing, rash, fever
Donepezil	Acetylcholinesterase inhibitor	Nausea, muscle cramps, fatigue, weight loss, vomiting
Galantamine	Acetylcholinesterase inhibitor	Nausea, diarrhea, vomiting, loss of appetite, headache
lecanemab	Monoclonal antibody for aggregated Aβ	Brain swelling, ARIA, headache, falls
Memantine	N-methyl D-aspartate antagonist	Headache, dizziness, diarrhea, constipation, confusion
Rivastigmine	Acetylcholinesterase inhibitor	Nausea, diarrhea, weight loss, vomiting, indigestion, muscle weakness

## Data Availability

Not applicable.
